# Diacetoxyscirpenol as a new anticancer agent to target hypoxia-inducible factor 1

**DOI:** 10.18632/oncotarget.11529

**Published:** 2016-08-23

**Authors:** Yong-Joon Choi, Hyun-Woo Shin, Yang-Sook Chun, Alain Simplice Leutou, Byeng Wha Son, Jong-Wan Park

**Affiliations:** ^1^ Department of Biomedical Science, Seoul National University College of Medicine, Seoul, 110-799, Republic of Korea; ^2^ Department of Pharmacology, Seoul National University College of Medicine, Seoul, 110-799, Republic of Korea; ^3^ Ischemic/Hypoxic Disease Institute, Seoul National University College of Medicine, Seoul, 110-799, Republic of Korea; ^4^ Cancer Research Institute, Seoul National University College of Medicine, Seoul, 110-799, Republic of Korea; ^5^ Department of Chemistry, Pukyong National University, Busan, 608-737, Republic of Korea

**Keywords:** cancer, hypoxia, HIF-1, drug screening, diacetoxyscirpenol

## Abstract

Hypoxia activates hypoxia-inducible factor 1, which promotes the progression of malignancy by stimulating angiogenesis and by augmenting the ability of tumors to survive. Thus, HIF-1 is one of the most compelling targets for treating cancers. The aim of this study was to find a small molecule that inhibits HIF-1 under hypoxia in cancer cells. 7,280 compounds in a chemical library were tested in a cancer cell line expressing luciferase HIF-dependently. Through three rounds of screening, we finally picked up a compound that originates from a marine bacterium parasitizing red alga. The antibiotic potently inhibited HIF-1 expression and its transcriptional activity in cancer cells exposed to hypoxia. Through two-step fractionation, diacetoxyscirpenol was purified and identified as a HIF-inhibiting ingredient. Mechanistically, diacetoxyscirpenol inhibits the synthesis of HIF-1α protein and also interferes with the dimerization of HIF-1α and ARNT. It attenuates HIF-mediated gene expression in cancer cells exposed to hypoxia, and by doing so reduces tumorigenic and angiogenic potentials of cancer cells. More importantly, diacetoxyscirpenol retarded tumor growth in mice, and reduced HIF-1α expression and vascular formation in the tumors. Overall, diacetoxyscirpenol is considered a potential drug deregulating the HIF-1 signaling pathway, and it could be beneficially employed for treating malignant tumors with hypoxic microenvironment.

## INTRODUCTION

Hypoxia is a common feature in solid tumors and plays crucial roles for tumor progression and angiogenesis [1, 2]. To survive under a hypoxic microenvironment, cancer cells require the activation of many genes essential for their adaptation to the condition, which is mainly driven by the transcription factor HIF-1 (hypoxia-inducible factor 1) [3]. HIF-1 is a heterodimeric complex composed of HIF-1α and ARNT (aryl hydrocarbon receptor nuclear translocator), both of which belong to the basic helix-loop-helix PAS family [4]. HIF-1α is a hypoxia-inducible protein that plays a primary role in gene expression and ARNT is a constitutive protein that assists HIF-α bind to DNA. Briefly reviewing the oxygen-dependent regulation of HIF-1α, HIF-1α is hydroxylated oxygen-dependently at two proline residues under normoxia, which triggers the ubiquitination and proteasomal degradation of HIF-1α. When the ambient oxygen content drops below the level critical for the prolyl-hydroxylation, HIF-1α is stabilized and enters into the nucleus, where it targets 100 or more genes [5–7].

To date, the roles of HIF-1 in tumor progression have been intensively explored and HIF-1α is generally regarded as a tumor promoting factor. The expression of HIF-1 in tumor specimens positively correlates with tumor aggressiveness and poor prognosis [8]. Also, many tumor grafting studies have supported the positive effects of HIF-1 on tumorigenesis, tumor growth, and tumor invasion [9]. Given clinical and experimental evidence, HIF-1 has been believed as a promising target for treating tumors. Indeed, many types of HIF-1 inhibitors have been discovered, and some of them are currently under clinical trials. HIF-1 inhibitors act through various mode-of-actions as followings. Bortezomib, nutlin-3, and echinomycin functionally inhibit the HIF-1-mediated gene expressions [10–12]. HSP90 inhibitors, microtubule disruptors, and YC-1 destabilize HIF-1α in the post-translational level [13–15]. Several agents, such as topotecan, digoxin, PX-478, rapamycin, and chaetocin, have been reported to block de novo synthesis of HIF-1α protein [16–20].

The aim of this study was to find a small molecule that inhibits HIF-1 under hypoxia in cancer cells. A human hepatoma cell line, which stably expresses the luciferase reporter gene targeted by HIF-1, was established and used for searching for HIF-1 inhibitors in a chemical library containing 7,280 compounds. Finally, we picked up a compound that originates from a marine bacterium living on red alga, and identified this compound as diacetoxyscirpenol. Moreover, we examined how the compound inhibits HIF-1 and whether it has anti-cancer and anti-angiogenic activities.

## RESULTS

### Screening of HIF-1 inhibitors

**Figure 1:**
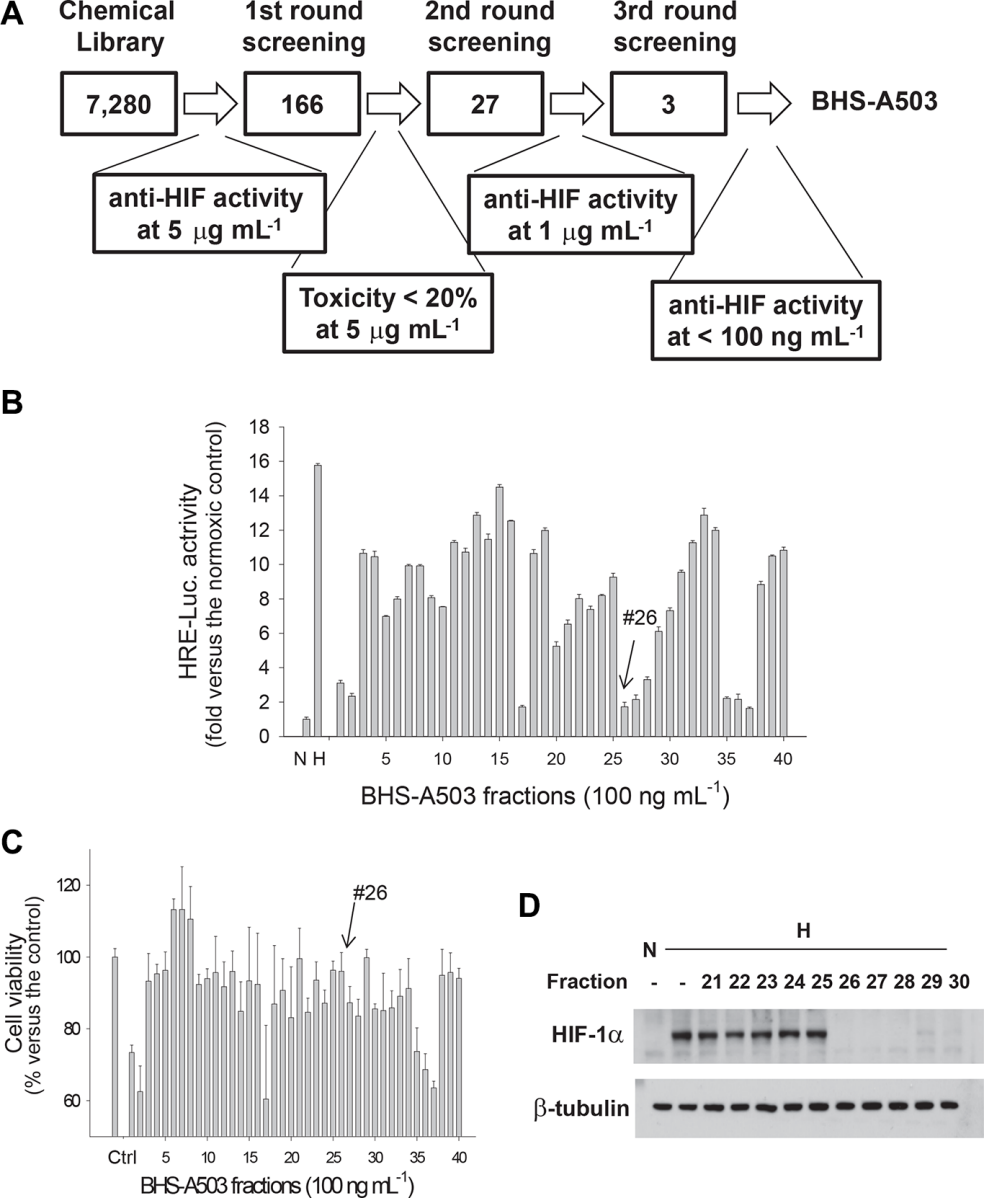
The 1^st^ round screening of anti-HIF compounds in BHS-A503 (**A**) Scheme for searching for compounds having anti-HIF activity. (**B**) Effects of BHS-A503 fractions against HIF-driven transcription. Hep3B cells harboring the EPO enhancer/luciferase reporter were treated with one of 40 fractions (100 ng·mL^−1^) form BHS-A503 for 4 hours, and then incubated at 1% oxygen for 16 hours. Luciferase activities in cell lysates were measured using a luminometer. (**C**) Effects of BHS-A503 fractions on cell viability. Hep3B cells were treated with 100 ng/mL of each fraction for 24 hours, and cell viabilities were evaluated by MTT assay. (**D**) Effects of BHS-A503 fractions on HIF-1α expression. Hep3B cells were treated with 100 ng·mL^−1^ of each fraction under hypoxia for 12 hours. HIF-1α and β-tubulin levels were analyzed by immunoblotting. All results were obtained from 4 independent experiments and each bar represents the mean + standard deviation.

We established the stable Hep3B cell line that expresses luciferase HIF-1-dependently under hypoxia and searched for anti-HIF-1 compounds, as was summarized in Figure 1A. We first checked chemiluminescence emitting form the cells which had been incubated at 1% oxygen with DMSO or each (finally 5 μg·mL^−1^) of 7,280 compounds in a chemical library for 16 hours. From the first-round screening, we picked up 166 candidates. To rule out the possibility that luciferase expression is non-specifically impaired due to toxicity, we checked whether the candidates at 5 mg·mL^−1^ were not toxic (less than 20% cell death) using MTT analysis. From the second-round screening, 27 compound were selected as HIF-1 inhibitors. To further search for potent inhibitors, we reevaluated the anti-HIF-1 effects of 27 candidates at a concentration of 1 mg·mL^−1^ and picked up three candidates. Finally, we selected the most potent compound “BHS-A503” that inhibited HIF-1 at < 100 ng·mL^−1^. The compound was an unrefined extract from *Bacillus licheniformis*, which is parasitic on red alga *Gelidium pacificum.*

### Purification and identification of a HIF-1 inhibitor from the BHS-A503 fraction

**Figure 2:**
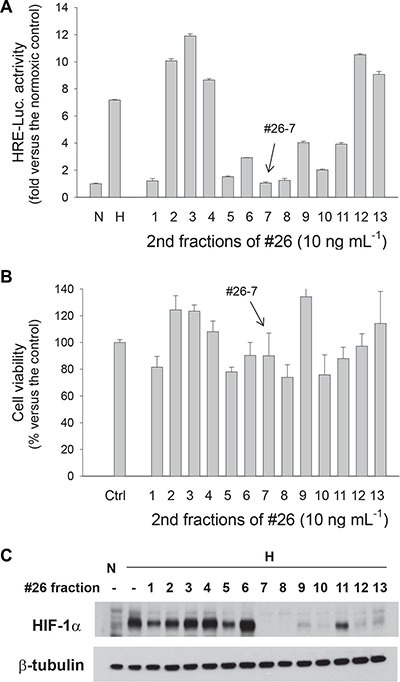
The 2^nd^ round screening of anti-HIF compounds in BHS-A503 #26 fraction (**A**) Effects of #26 fractions against HIF-driven transcription. The EPO enhancer/luciferase-harboring Hep3B cells were treated with one of 13 fractions (10 ng·mL^−1^) from BHS-A503 #26 for 4 hours, and then incubated under hypoxia for 16 hours. Luciferase activities in cell lysates were measured using a luminometer. (**B**) Effects of BHS-A503 #26 fractions on cell viability. Hep3B cells were treated with 10 ng·mL^−1^ of each fraction for 24 hours, and cell viabilities were evaluated by MTT assay. (**C**) Effects of BHS-A503 #26 fractions on HIF-1α expression. Hep3B cells were treated with 10 ng·mL^−1^ of each fraction under hypoxia for 12 hours. HIF-1α and β-tubulin levels were analyzed by immunoblotting. All results were obtained from 4 independent experiments and each bar represents the mean and standard deviation.

To purify a single compound from the bacterial extract, we performed silica-gel flash chromatography and separated the extract into 40 fractions. Of them, we picked up the fraction 26 because it inhibited HIF-1 activity by > 85% (Figure 1B) and was also not significantly toxic (Figure 1C). The effects of the fractions against HIF-1 were rechecked by evaluating the HIF-1α protein level using Western blotting. As was expected, HIF-1α protein was not detected under normoxia but was notably induced under hypoxia. We also found that the fraction 26 at 100 ng·mL^−1^ attenuated the hypoxic induction of HIF-1α almost completely (Figure 1D). Since the fraction 26 was not a single compound, we further separated the fraction 26 into 13 fractions using LH-20 column chromatography. Consequently, the 7^th^ fraction of the fraction 26, which is designated #26-7, was identified not only to inhibit HIF-1 activity even at 10 ng·mL^−1^ but also to be less toxic (Figure 2A and 2B). A Western blotting showed that #26-7 suppressed HIF-1α expression very effectively (Figure 2C).

### Anti-HIF effects of #26-7 in different cancer cell lines

**Figure 3:**
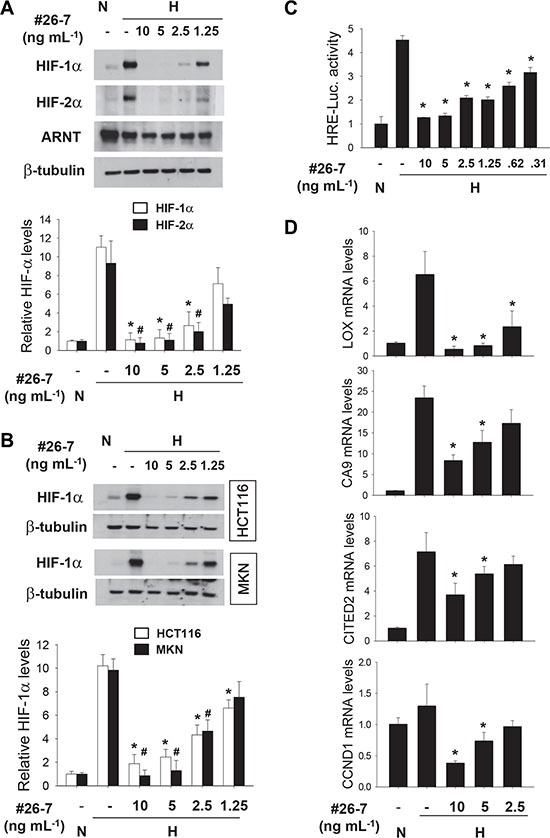
Anti-HIF activity of compound #26-7 (**A** and **B**) A549, HCT116, or MKN cells were pre-treated with #26-7 for 4 hours, and further incubated under hypoxia for 8 hours. Protein levels were analyzed by immunoblotting (top). Each bar represents the mean and standard deviation (*n* = 3), and *and # denote *P* < 0.05 versus the hypoxic control (bottom). (**C**) A549 cells, which had been co-transfected with the EPO enhancer-luciferase and the CMV promoter-galactosidase plasmids (1 mg of each), were treated with #26-7 for 4 hours, and then incubated under hypoxia for 16 hours. Luciferase activities were normalized to galactosidase activities, which were presented as relative values to the normoxic control level. Each bar represents the mean + standard deviation (*n* = 4) and *denotes *P* < 0.05 versus the hypoxic control (the 2^nd^ bar). (**D**) Quantitative RT-PCR was performed to check the expression of HIF-targeted genes in A549 cells which were treated with #26-7 under normoxia (N) or hypoxia (H) for 16 hours. Each assay was done in triplicate and the result was divided by β-actin level in the corresponding sample. Each bar represents the mean and standard deviation (*n* = 4) and *denotes *P* < 0.05 versus the hypoxic control.

**Figure 4:**
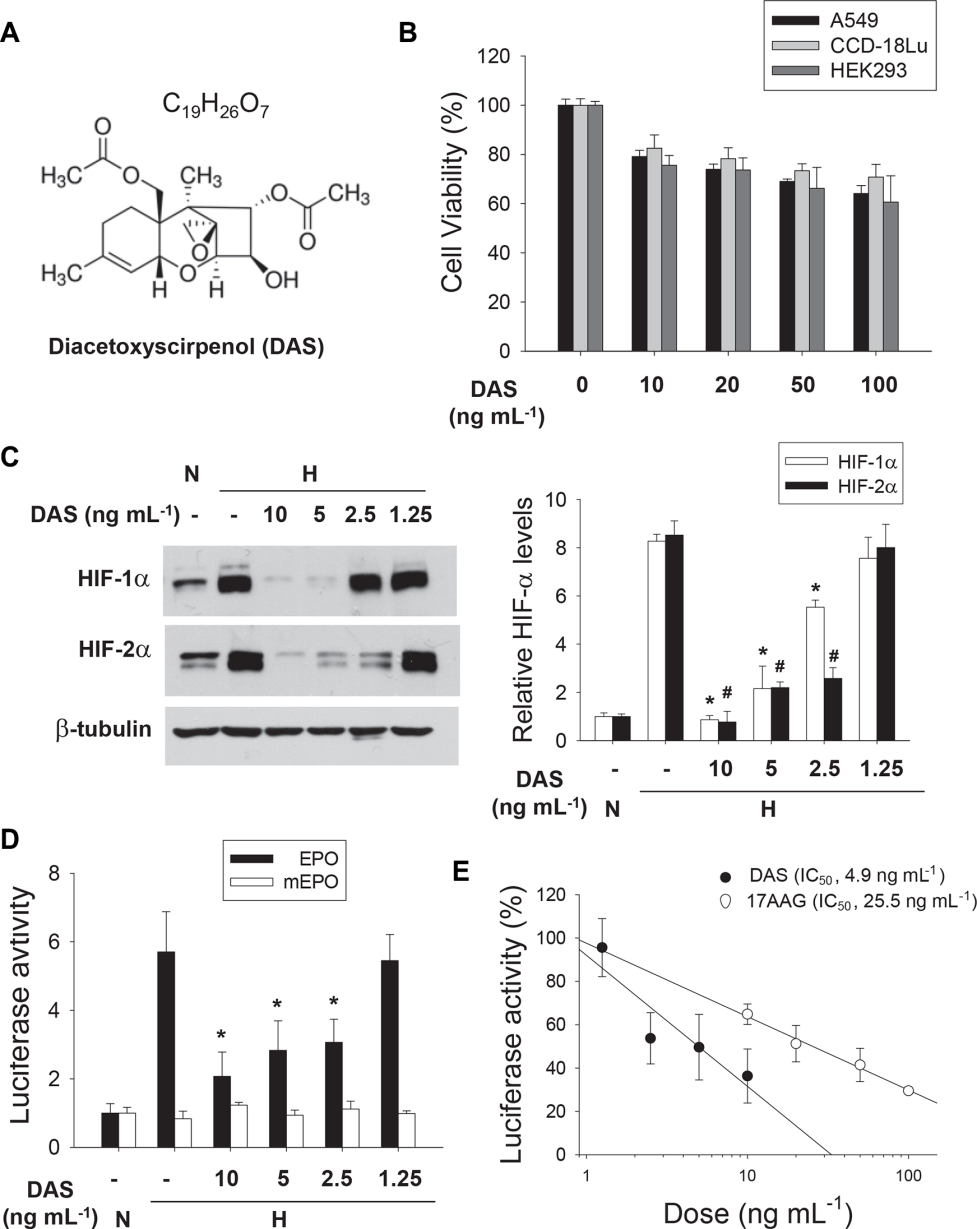
Identification of BHS-A503 #26-7 (**A**) Compound #26-7 was identified as diacetoxyscirpenol (DAS) in [α]_D_, LR-EI-MS, and NMR (^1^H, ^13^C, DEPT) analyses. (**B**) Comparison of toxicities of DAS among cancer cell and normal cells. A549, CCD-18Lu, and HEK293 were treated with the indicated doses of DAS for 24 hours. Cell viability was evaluated by MTT assay, and each bar represents the mean + standard deviation from 3 independent experiments. (**C**) A549 cells were treated with 1.25–10 ng·mL^−1^ of DAS, which was purchased from a company, for 4 hours and subjected to 8 hour-hypoxia. Protein levels were analyzed by immunoblotting (top). Each bar represents the mean and standard deviation (*n* = 3), and *and # denote *P* < 0.05 versus the hypoxic control (bottom). (**D**) A549 cells, which had been co-transfected with EPO enhancer-luciferase and galactosidase plasmids (1 mg of each), were treated with DAS for 4 hours and incubated under hypoxia for 16 hours. Luciferase activities were normalized to galactosidase activities and are presented as relative values to the normoxic control. Each bar represents the mean and standard deviation (*n* = 4) and *denotes *p* < 0.05 versus the hypoxic control. (**E**) A549 cells harboring the EPO-Luc plasmid were treated with DAS or 17AAG at the indicated concentrations for 4 hours, and incubated under hypoxia for 16 hours. Luciferase activities in drug-treated groups were divided by those in drug-free group. Each bar represents the mean and standard deviation (*n* = 4), and the half maximal inhibitory concentration (IC_50_) was calculated using the ED50plus program.

We next examined whether #26-7 has anti-HIF activity in cancer cells other than Hep3B. #26-7 suppressed HIF-1α expression in a dose-dependent manner, whereas it did not substantially affect ARNT expression (Figure 3A, the 1^st^ and 2^nd^ rows). Likewise, #26-7 also effectively suppressed HIF-1α expression in HCT116 and MKN cells (Figure 3B). Apparently, the efficacy of #26-7 on HIF-1α inhibition appears to be similar among three cell lines. In the EPO enhancer reporter assay, #26-7 was found to inhibit the HIF-1 driven transcription under hypoxia (Figure 3C). To examine whether #26-7 regulates the cellular response to hypoxia, we analyzed mRNAs of the HIF target genes in A549 cells. The hypoxic induction of LOX, CA9, CyclinD1, and CITED2 mRNAs was substantially attenuated by #26-7 (Figure 3D). Using [α]_D_, LR-EI-MS, and NMR (^1^H, ^13^C, DEPT) analyses, #26-7 was identify as diacetoxyscirpenol [21]. Its structure is illustrated in Figure 4A. First, we compared the toxicity of the purchased diacetoxyscirpenol in A549 and two normal cell-lines. Even at the higher concentrations of diacetoxyscirpenol, no significant differences in toxicity were observed among the cell-lines (Figure 4B). Given that this reagent also inhibited the expression and activity of HIF-1/2α (Figure 4C and 4D), diacetoxyscirpenol was considered an HIF-inhibiting ingredient included in #26-7. Compared with a known HIF-1 inhibitor 17AAG, diacetoxyscirpenol was shown to be more potent in HIF-1 inhibition (Figure 4E).

### Diacetoxyscirpenol deregulates HIF-1 signaling in two different steps

**Figure 5:**
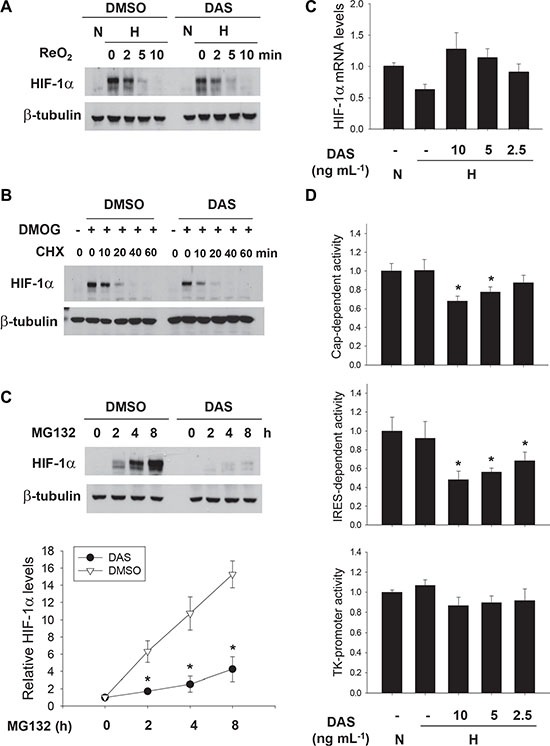
Diacetoxyscirpenol downregulates HIF-1α in the translational level (**A**) Effect of DAS on oxygen-dependent degradation of HIF-1α. A549 cells were incubated with DMSO or DAS under hypoxia for 12 hours. Following re-oxygenation for the indicated periods (0 to 10 minutes), cells were lysed and HIF-1α levels were analyzed by immunoblotting. (**B**) After treated with 500 μM DMOG for 8 hours, A549 cells were pre-incubated with DMSO or DAS (10 ng·mL^−1^) for 1 hour, and then treated with 10 mg·mL^−1^ of CHX for the indicated times. HIF-1α levels were determined by immunoblotting. (**C**) Effect of DAS on de novo synthesis of HIF-1α protein. After treated with DMSO or DAS 4 hours, A549 cells were additionally treated with 10 μM MG132 to induce the accumulation of newly synthesized HIF-1α. HIF-1α levels were determined at the indicated times (0 to 8 hours) by immunoblotting (top). Each bar represents the mean and standard deviation (*n* = 3), and *denotes *P* < 0.05 versus the DMSO control at the corresponding time (bottom). (**D**) Effect of DAS on the transcription of the HIF-1α gene. Quantitative RT-PCR was performed to check the HIF-1α mRNA level in A549 cells which were treated with DAS for 16 hours. Each assay was done in triplicate and the result was divided by β-actin level in the corresponding sample. Each bar represents the mean and standard deviation (*n* = 4). (D) Effect of DAS on the 5′UTR-driven translation of HIF-1α. Cap-dependent and IRES-dependent translational rates of HIF-1α were analyzed using TK promoter-5′UTR-luciferase and CMV promoter-GFP-5′UTR-luciferase reporters, respectively. The TK promoter-luciferase vector was used as a background control. A549 cells, which had been co-transfected with one of these reporters and galactosidase plasmids, were subjected to 16 hour-hypoxia in the absence or presence of DAS. Luciferase activities were normalized to galactosidase activities and are presented as relative values to the normoxic control. Each bar represents the mean and standard deviation (*n* = 4) and *denotes *p* < 0.01 versus the hypoxic control.

To understand how diacetoxyscirpenol downregulates HIF-1α, we first checked the stability of HIF-1α protein. HIF-1α degradation was induced by reoxygenation after the protein was accumulated under hypoxia (Figure 5A) or by cycloheximide treatment after it was stabilized using a HIF prolyl hydroxylase inhibitor, dimethyloxaloylglycine (DMOG) (Figure 5B). However, the rate of HIF-1α degradation was not facilitated in the presence of diacetoxyscirpenol, suggesting no effect of diacetoxyscirpenol on HIF-1α stability. Next, we analyzed de novo synthesis of HIF-1α using MG132, which can induce the accumulation of newly synthesized HIF-1α protein by blocking protein degradation. Strikingly, the synthesis of HIF-1α protein was abolished by diacetoxyscirpenol (Figure 5B). To further examine the mechanism underlying the blockade of HIF-1α synthesis, we measured the mRNA levels of HIF-1α, but diacetoxyscirpenol did not inhibit the transcription of HIF-1α (Figure 5C), which encouraged us to analyze the translation of HIF-1α. The translation of HIF-1α mRNA is initiated through two pathways, 5′ cap-dependent translation and internal ribosome entry site (IRES)-dependent translation, both of which are determined by the 5′-UTR of HIF-1α mRNA [22]. We analyzed the activities of two luciferase reporters reflecting each pathway of HIF-1α translation. Consequently, diacetoxyscirpenol inhibited both the pathways of HIF-1α translation, whereas it did not affect the activity of TK promoter without the 5′-UTR (Figure 5D). Diacetoxyscirpenol seems to inhibit de novo synthesis of HIF-1α protein by blocking the 5′-UTR-mediated translation of HIF-1α mRNA.

**Figure 6:**
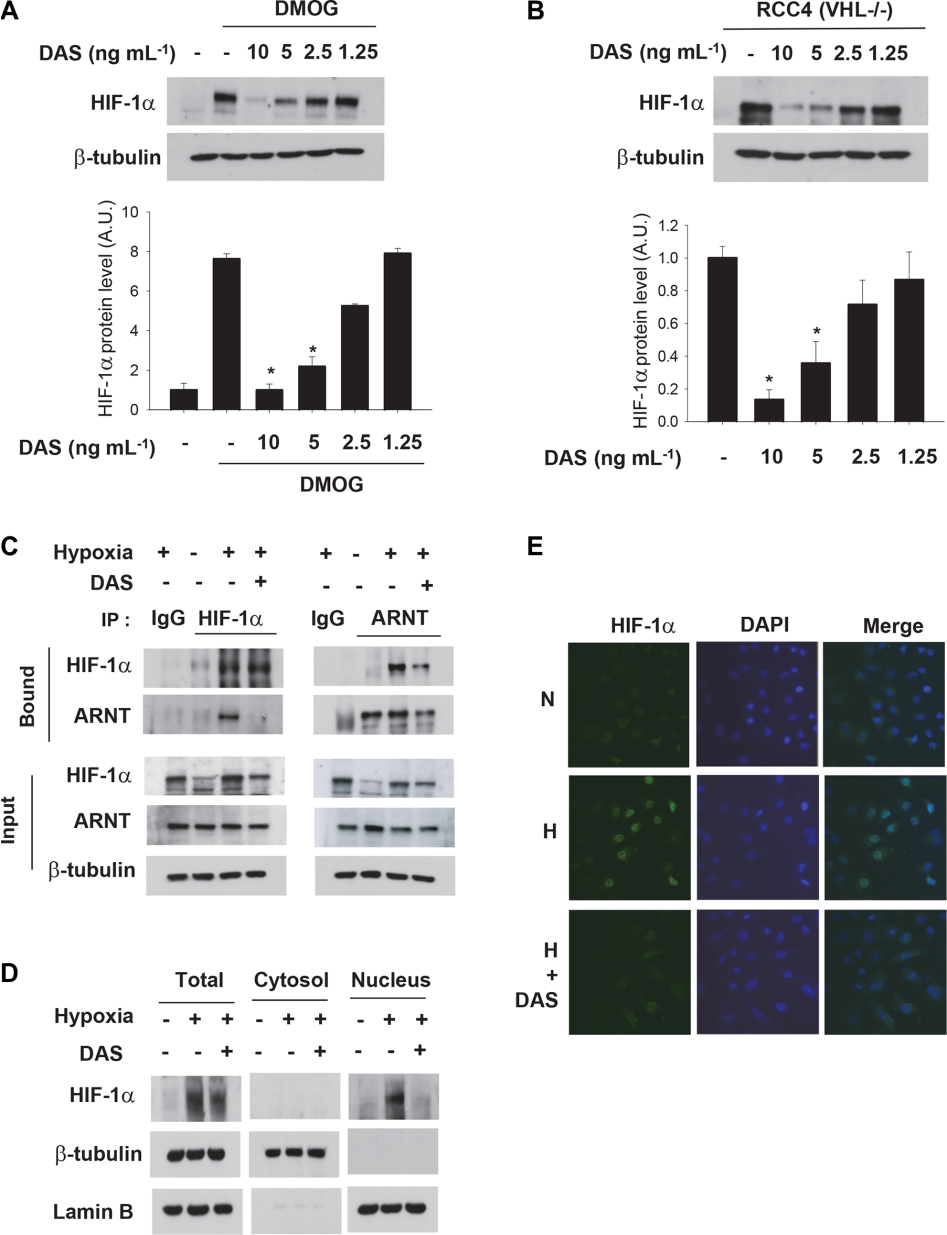
Diacetoxyscirpenol blocks the formation of the HIF-1 complex (**A**) *VHL*-null RCC4 cells were treated with DAS for 12 hours, and subjected to immunoblotting. (**B**) After treated with DAS for 4 hours, A549 cells were incubated with 0.5 mM DMOG to inhibit the hydroxylation of HIF-1α, and subjected to immunoblotting. (**C**) A549 cells were pretreated with DAS (5 ng·mL^−1^) for 4 hours, and further incubated under hypoxia for 8 hours. HIF-1α or ARNT were precipitated with the indicated antibodies, and then, the precipitates was subjected to immunoblotting. The specificity of immunoprecipitation was verified using non-immunized serum (IgG). (**D**) The lysates of A549 cells, which had been treated as indicated, were separated into the cytosolic and nuclear fractions, which were subjected to immunoblotting. Lamin B and β-tubulin were analyzed to verify the specificity of each fraction. (**E**) A549 cells were treated DMSO or DAS (5 ng·mL^−1^) under normoxic or hypoxic conditions for 16 hours. The cells were co-stained with anti-HIF-1α antibody (green) and DAPI (blue). The fluorescence images were captured at ×400 magnification under a fluorescent microscope.

To rule out other actions of diacetoxyscirpenol on oxygen-dependent HIF-1 regulation, we examined if it affects the HIF-1α hydroxylation by PHD enzymes or the HIF-1α ubiquitination/degradation by the pVHL-E3 complex. However, diacetoxyscirpenol suppressed HIF-1α expression even in the presence of DMOG and also in VHL-null RCC4 cells (Figure 6A and 6B). These findings further support our notion that diacetoxyscirpenol does not affect the degradation process of HIF-1α. To get its functionality as a transcription factor, HIF-1α should dimerize with ARNT in the nucleus. Interestingly, diacetoxyscirpenol interfered with the dimerization of HIF-1α and ARNT (Figure 6C), which may be attributed to impaired nuclear translocation of HIF-1α. The level of nuclear HIF-1α was cross-checked using immunoblotting (Figure 6D) and immunofluorescence (Figure 6E). Collectively, diacetoxyscirpenol seem to attenuate HIF-1 signaling in two different ways.

### Diacetoxyscirpenol inhibits anchorage-independent cancer growth and angiogenesis

**Figure 7:**
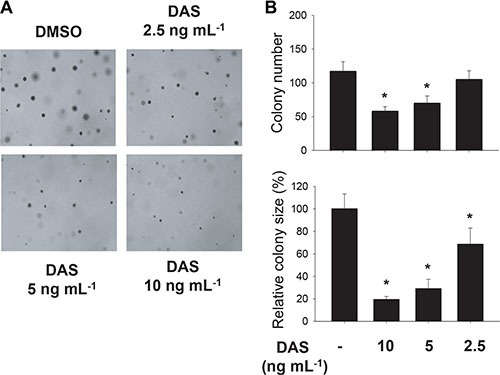
Effect of diacetoxyscirpenol on the colonial growth of A549 cancer cells (**A**) After treated with DAS twice a week for 3 weeks, A549 cells in the top agar were stained with 0.05% crystal violet. (**B**) Colony size was measured using ImageJ and colonies whose sizes were over 0.2 mm^2^ were counted. Each bar represents the mean + standard deviation (*n* = 4) and *denotes *P* < 0.05 versus the DMSO control.

**Figure 8:**
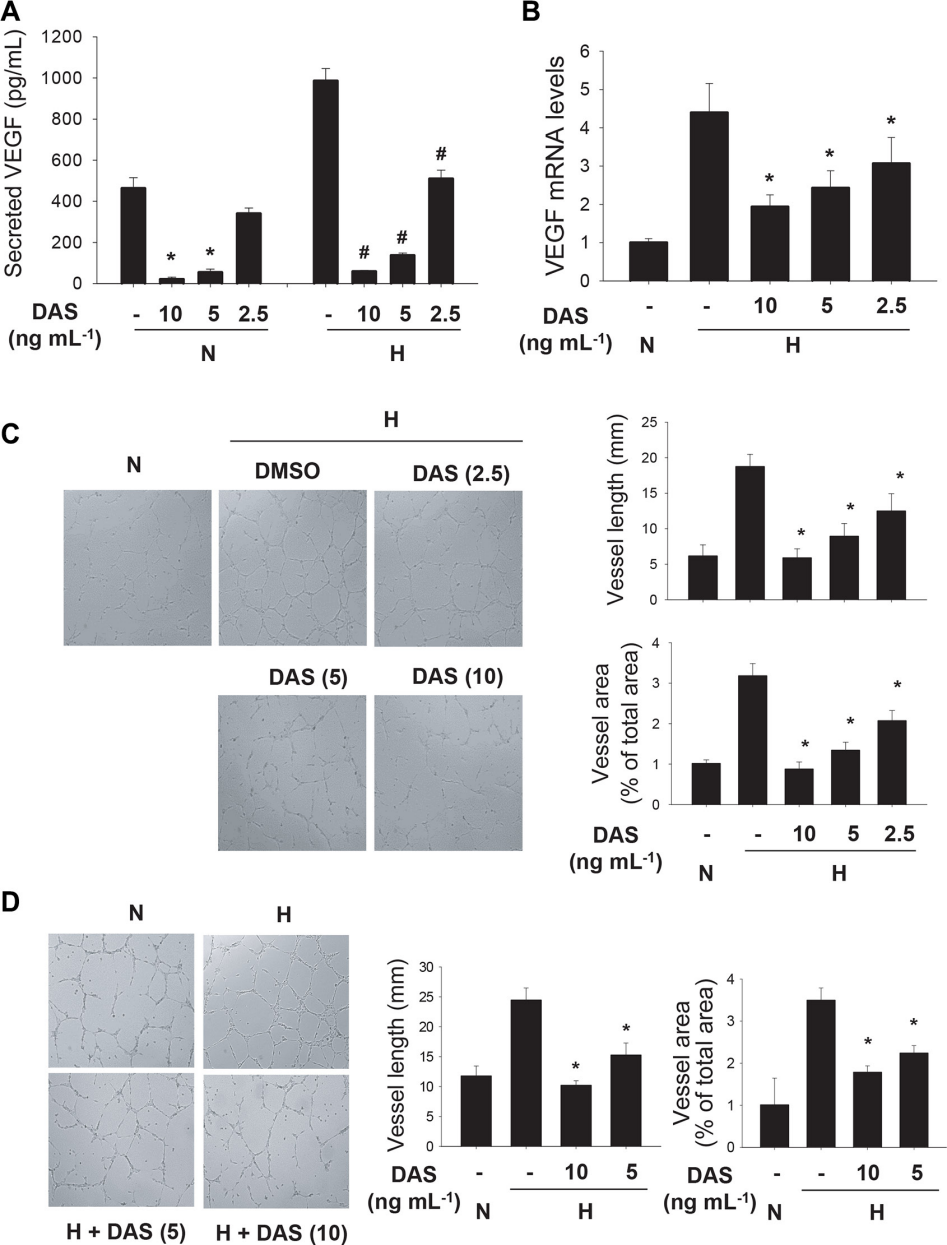
Anti-angiogenic effect of diacetoxyscirpenol (**A**) The conditioned media were collected from A549 cells which had been incubated with DAS under normoxic or hypoxic conditions. The concentrations (means + standard deviation, *n* = 4) of VEGF in the conditioned media were measured using an ELISA kit. *and # denote *P* < 0.05 versus the normoxic control and the hypoxic control, respectively. (**B**) After A549 cells were treated with DAS for 24 hours under hypoxia, the VEGF mRNA levels were analyzed by quantitative RT-PCR. Each assay was done in triplicate and the VEGF level was divided by β-actin level in the corresponding sample. Each bar presents the mean + standard deviation (*n* = 4) of relative values and *denotes *P* < 0.05 versus the hypoxic control. (**C**) HUVECs were cultured on Matrigel™-coated plates for 16 hours in the conditioned media from A549 cells which had been treated with DAS for 24 hours under normoxic or hypoxic conditions. The reorganization of HUVECs was pictured under an inverted microscope (left). Quantitative analyses of tube lengths (mm) and tube area (% of total area) were performed using ImageJ (right). (**D**) After A549 cells were incubated for 24 hours under normoxic or hypoxic conditions, conditioned media were collected and applied to HUVECs in Matrigel™-coated plates. HUVECs were further incubated with DMSO or DAS for 16 hours. HUVECs were photographed (left) and endothelial tubes were quantified using ImageJ (right). Each bar presents the mean + standard deviation (*n* = 4) and *denotes *P* < 0.05 versus the hypoxic control.

Anchorage-independent cell growth is a representative property of cancer cells with tumorigenic potential, and it has been reported to occur depending on HIF-1α [23, 24]. Therefore, we examined if diacetoxyscirpenol can negate the tumorigenic potential of A549 cells using a colony formation assay. At a glance, colonies of A549 were shown at a lesser density in the presence of diacetoxyscirpenol (Figure 7A). Both the number and size of colonies were significantly reduced by diacetoxyscirpenol in a dose-dependent manner (Figure 7B). We next investigated if diacetoxyscirpenol attenuates the angiogenic potential of cancer cells because HIF-1α plays a key role in tumor angiogenesis [2, 25]. Diacetoxyscirpenol substantially inhibited VEGF secretion from A549 cells in either normoxic or hypoxic conditions (Figure 8A). As expected, diacetoxyscirpenol attenuated the hypoxic induction of VEGF mRNA (Figure 8B). To check the angiogenic potential of A549 cells, tube formation of HUVECs was analyzed in the presence of A549-driven conditioned media. Endothelial tube formation was augmented by the conditioned media from A549 cells exposed to hypoxia. However, in the conditioned media from A549 cells treated with diacetoxyscirpenol, the tube formation was significantly reduced (Figure 8C). To test the possibility that diacetoxyscirpenol *per se* inhibits the endothelial tube formation, the compound was separately administered into the endothelial culture plates containing conditioned media from hypoxic A549 cells. Given that the tube formation was attenuated by diacetoxyscirpenol (Figure 8D), diacetoxyscirpenol is also likely to have a direct effect against vascular formation. These results suggest that diacetoxyscirpenol inhibits tumorigenic and angiogenic potentials of cancer cells under hypoxia.

### Diacetoxyscirpenol inhibits the growth of lung carcinoma grafts in mice

**Figure 9:**
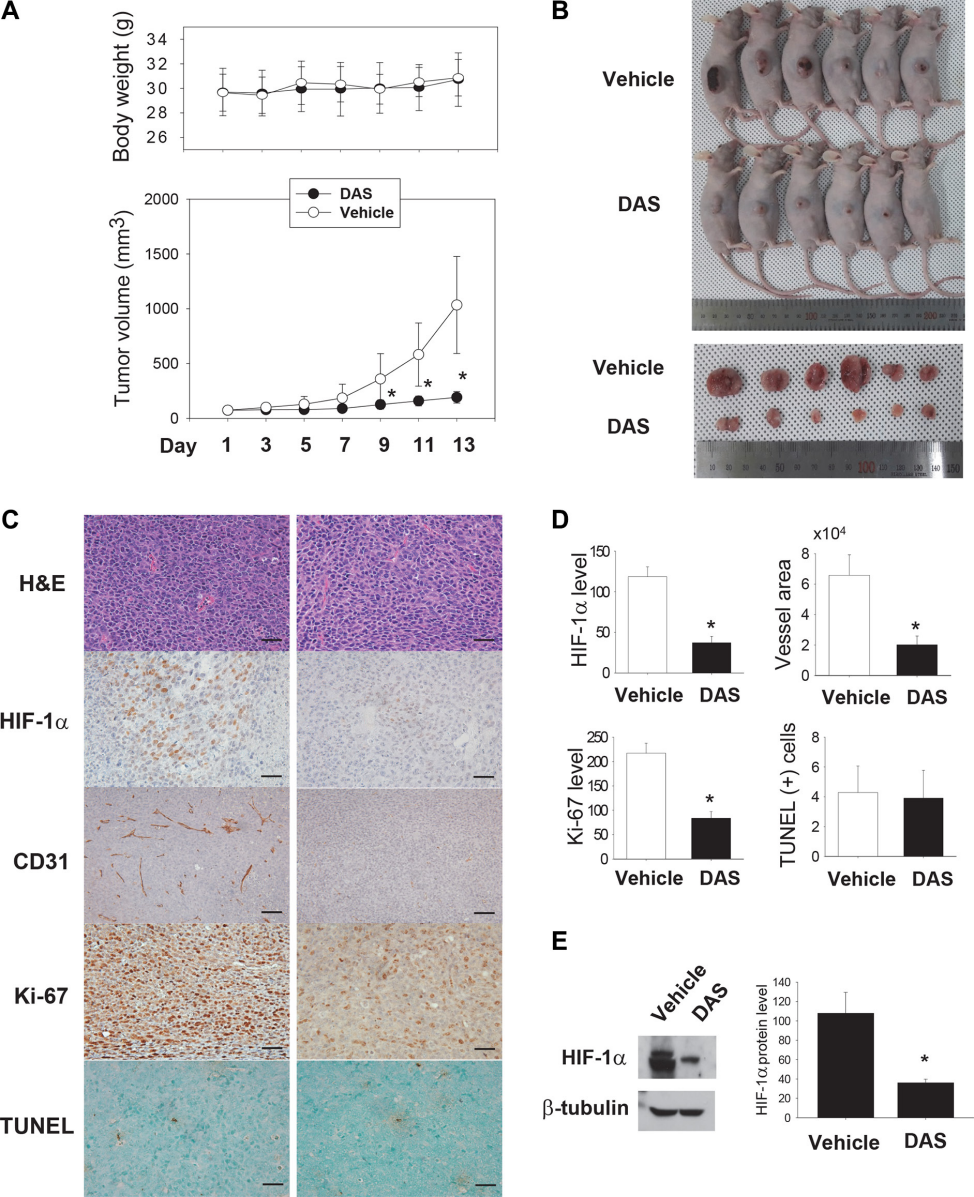
Anticancer effect of diacetoxyscirpenol (**A**) Tumor growth curves. LCC1 cells were grafted on the flanks of mice, and the tumor-bearing mice were injected daily with vehicle (*n* = 6) or 1 mg·kg^−1^ of DAS (*n* = 6) for 2 weeks. Each circle represents the mean and standard deviation of body weight (top) or tumor volume (bottom). *denotes *P* < 0.05 vs. the vehicle group. (**B**) Gross appearance of tumor-bearing mice or excised tumors. (**C**) Immunohistochemical analyses. Excised tumors were fixed, paraffin-embedded, and cut into 4 mm sections. The specimens were stained with hematoxylin and eosin (H&E), or immunostained with anti-HIF-1α, anti-CD31 and anti-Ki-67 antibodies. TUNEL staining was performed to detect cancer cells undergoing apoptosis. (**D**) Four fields (x400 magnification) per section and four sections of each tumor were reviewed for histologic assessments. HIF-1α, CD31, Ki-67, TUNEL-positive cells were counted, and the areas of CD31-positive vessel were measured using the Image J. Each bar represents the mean + standard deviation (*n* = 6) and *denotes *P* < 0.05 versus the vehicle group. Scale bar = 100 mm. (**E**) In tumor homogenates, HIF-1α protein was immunoblotted (left), its level was analyzed using ImageJ (right). Each bar represents the mean + standard deviation (*n* = 5) and *denotes *P* < 0.05 versus the vehicle control.

To *in vivo* examine the anticancer activity of diacetoxyscirpenol, this agent was administered into mice bearing Lewis lung carcinoma. When tumor volume reached about 100 mm^3^, mice were intraperitoneally injected with DMSO (vehicle) or diacetoxyscirpenol. No substantial changes in gross appearance and body weight suggest that diacetoxyscirpenol at the used dose is not highly toxic (Figure 9A). Diacetoxyscirpenol retarded tumor growth (Figure 9A), and excised tumors were noticeably smaller in diacetoxyscirpenol-treated mice than in vehicle-treated mice (Figure 9B). HIF-1α and Ki-67 (a marker for cell proliferation) were less expressed, and vessels were poorly developed in tumor specimens from diacetoxyscirpenol-treated mice (Figure 9C and 9D). The HIF-1α suppression by diacetoxyscirpenol was confirmed by immunoblotting HIF-1α in tumor homogenates (Figure 9E). These results suggest that diacetoxyscirpenol attenuates cancer cell proliferation by impairing tumor adaptation to hypoxic microenvironment. However, given that the numbers of TUNEL-stained apoptotic cells were not different between two groups, this agent may not kill cancer cells directly.

## DISCUSSION

To find a new anticancer small molecule to target HIF-1, we performed the high-throughput screening using a luciferase reporter system reflecting HIF-driven transcription, and finally selected a marine bacterial extract based on high potency on HIF inhibition and less cytotoxicity. Through two fractionation processes of the extract, diacetoxyscirpenol was purified and identified as an active ingredient having anti-HIF activity. Mechanistically, diacetoxyscirpenol repressed HIF-1 by inhibiting either HIF-1α translation or its dimerization with ARNT. Furthermore, diacetoxyscirpenol blunted HIF-mediated induction of hypoxic genes, and by doing so attenuated anchorage-independent colony formation, endothelial tube formation, and tumor growth in mice. Therefore, we propose that diacetoxyscirpenol be a potential anticancer agent targeting HIF-1.

To search for compounds inhibiting the HIF-1 signaling pathway, we performed two different methods to evaluate this pathway, namely, the HRE-luciferase reporter and Western blotting for HIF-1α. However, as shown in Figure 1B and 1D and in Figure 2A and 2C, the results from the two analyses were somewhat miss-matched with each other. Actually, the functionality of HIF-1 is determined by diverse factors other than the amount of HIF-1α protein. For instance, HIF-1α is modified (i.e., hydroxylated, phosphorylated, sumoylated, and neddyated), and interacts with many proteins, thereby modulating the HIF-1 activity [26, 27]. The discrepancies between the results from two analyses may be attributed to the post-translation regulations of HIF-1α. Therefore, we chose the compounds that inhibited HIF-1 at both the analyses.

Diacetoxyscirpenol contains the 12,13-epoxytrichothecene group of sesquiterpenes as the core structure. It has been known as one of harmful metabolites produced from plant parasitic fungi belonging to *Fusarium* species [28]. Accidently, people or livestock have been subjected to food poisoning by diacetoxyscirpenol which contaminated agricultural crops, such as grains, potatoes, peas, soybeans [29]. However, diacetoxyscirpenol has been rarely reported to contaminate sea plants. Surprisingly, diacetoxyscirpenol was found to be also contained in a marine bacterium parasitizing red alga, suggesting that diacetoxyscirpenol probably cause food poisoning derived by sea foods. For keeping sea foods safe, it may be recommended to check the contamination of diacetoxyscirpenol.

Diacetoxyscirpenol and its analogues were developed as anti-cancer agents because it inhibited cell cycle progression and cell survival [30]. Diacetoxyscirpenol was also reported to potentiate the anti-cancer effect of cisplatin [31]. However, the clinical trial of diacetoxyscirpenol for cancer treatment was ceased at the phase II because it induced intolerable side effects, such as nausea, vomiting, fever, hypertension, and confusion [32, 33]. Nonetheless, we argue that diacetoxyscirpenol can be repositioned as an anti-HIF agent. Compared to the concentrations (> 100 nM) of diacetoxyscirpenol used to kill cancer cells directly [34], a lower concentration (< 5 nM) of diacetoxyscirpenol was found in our experiments to inhibit HIF-1. Indeed, cell viability was not substantially decreased by diacetoxyscirpenol at this concentration. If its dose is readjusted to a HIF-inhibiting level, diacetoxyscirpenol is expected to come out as a potential drug that specifically blocks hypoxia-triggered, malignant cancer behaviors, such as angiogenesis, metastasis, and drug resistance.

Diacetoxyscirpenol was shown to block the hypoxia-induced production of VEGF, which is a target gene of HIF-1. VEGF does not only drives angiogenesis but also acts as a cell survival factor, which is why VEGF inhibitors have been widely used as anti-cancer drugs. For example, bevacizumab interferes with VEGF binding to the VEGF receptors by sequestering VEGF, and inhibitors of VEGF receptor tyrosine kinase block the signaling pathway of the VEGF receptors [35, 36]. Diacetoxyscirpenol inhibited the VEGF secretion from cancer cells under hypoxia, thereby inhibiting the endothelial tube formation. Although it does not directly target endothelial cells, diacetoxyscirpenol may indirectly inhibit tumor-driven angiogenesis at least under hypoxic microenvironment.

Some anti-angiogenic agents, mostly in combination with conventional therapeutics, have been shown to have anticancer activity in preclinical and clinical studies [37]. However, the pros and cons of anti-angiogenic agents have been debated so far. In some preclinical studies, co-administration of anti-angiogenic agents was shown to aggravate tumor hypoxia and also to impair the delivery of cancer cell-targeting drugs, leading to acquired resistance to chemotherapy [38]. By contrast, beneficial actions of anti-angiogenic agents in combination therapies have been also suggested. These agents may improve oxygen and drug delivery within tumors through normalization of irregular tumor vessels [39]. Since the anti-angiogenic action of diacetoxyscirpenol may contribute to its anticancer effect, this conflicting point should be considered in developing diacetoxyscirpenol as an adjuvant cancer drug.

In conclusion, we found that diacetoxyscirpenol deregulates tumor adaptation to hypoxia by deregulating HIF-1α. To our best knowledge, diacetoxyscirpenol is for the first time identified to inhibit HIF-1. More importantly, it is possible that diacetoxyscirpenol is used as a HIF-1 inhibitor at a lesser dose than its cytotoxic dose. If a low dose of diacetoxyscirpenol is limitedly used for the purpose of preventing HIF-mediated tumor progression or drug resistance, it may be worthwhile reevaluating this agent as an adjuvant cancer drug.

## MATERIALS AND METHODS

### Cell culture and transfection

Hep3B (hepatoma), A549 (lung cancer), HCT116 (colon cancer), MKN (gastric cancer), RCC4/VHL−/− (renal cancer), and LLC1 (mouse lung cancer) cell lines were obtained from the American Type Culture Collection (Manassas, VA). The cells were cultured in Minimum Essential Media, Dulbecco's Modified Eagle Medium, or RPMI-1640, supplemented with 10% heat-inactivated FBS. Cells were incubated in a humidified atmosphere at 37°C under 20% O_2_/5% CO_2_ for normoxia or 1% O_2_/5% CO_2_ hypoxia. For transient transfection, cells at about 40% confluence were transfected with plasmids using Lipofectamin 2000 (Life Technologies, Carlsbad, CA). Cells were allowed to stabilize for 48 hours before being used in experiments.

### Reagents and antibodies

Diacetoxyscirpenol was purchased from LKT laboratories (Saint Paul, MN), and 17AAG and DMOG from Sigma-Aldrich Corp (St Louis, MO). Anti-HIF-2α (#7096) antibody was purchased from Cell Signaling Technology (Danvers, MA), and antibodies against ARNT (sc-8076), β-tubulin (sc-9104) and lamin-B (sc-6216) from Santa Cruz Biotechnology (Santa Cruz, CA). Anti-HIF-1α antiserum was generated in rabbits against a bacterially expressed fragment encompassing amino acids 418 to 698 of human HIF-1α, as described previously [40].

### Chemical library and fractionation of marine bacterial metabolites

A chemical library containing 7,280 compounds was kindly provided by Korea Chemical Bank (Daejeon, Korea; http://www.chembank.org/). A bacterial strain *Bacillus licheniformis* was isolated from the marine red alga *Gelidium pacificum* collected at Geomun Island in South Korea, and identified based on 18S rRNA analyses (performed in SolGent Co., Ltd., Daejeon, Korea). The bacterium was cultured in 20 L of culture medium consisting of soytone (0.1%), soluble starch (1.0%), and seawater (100%). The cultures were incubated at 29°C for 20 days on the static condition. The whole culture was filtered through cheesecloth to yield broth and mycelium residue, and the resulted broth were extracted with EtOAc to afford broth extract (1.1 g). The broth extract was subjected to silica gel flash column chromatography. Elution was performed with n-hexane-EtOAc (stepwise, 0–100% EtOAc) to yield eight fractions. Fraction 5 (15.5 mg) displayed HIF activity, and was further purified by silica gel flash column chromatography using n-hexane-EtOAc (stepwise, 10–100% EtOAc) to give crude diacetoxyscirpenol (8.0 mg), which was finally purified by LH-20 column chromatography (MeOH) to furnish diacetoxyscirpenol (1.5 mg).

### Reporter assays

To construct the EPO enhancer-driven luciferase reporter gene, the human EPO enhancer region (5-GGTACCGGCCCTACGTGCTGTCTCACACAGCCT GTCTGACCTCTCGACCTACCGGCCAGATCT-3) was inserted into the multi-cloning site of the pGL4 vector (Promega, Madison, WI). Mutated EPO luciferase was generated by altering the DNA sequence targeted by HIF-1 (CGTG to AAAA). Hep3B cells were transfected with 1 mg of the reporter plasmid using Lipofectamin 2000 (Life Technologies). After stabilized for 48 hours, the Hep3B cell lines stably harboring the reporter plasmid were selected under G418, and five stable cell lines were mixed to rule out the artifact generated by the integration of the plasmid into chromosomes. The cell line was pre-treated with each compound in a chemical library for 4 hours, and then incubated under normoxia or hypoxia for 16 hours. Cells were lysed and luciferase activity was measured using a LB960 luminometer (Berthold Technologies, Oak Ridge, TN). To evaluate the cap-dependent and the IRES-dependent translation of HIF-1α mRNA, we used thymidine kinase (TK) promoter/HIF-1α 5′-UTR/luciferase and CMV promoter/GFP/HIF-1α 5′-UTR/luciferase reporters, respectively [41]. The β-gal expression plasmid was cotransfected into cells to normalize transfection efficiency.

### Cell viability assays

An MTT labeling reagent (Sigma-Aldrich, St Louis, MI) was used to evaluate cytotoxicity. Cells were plated in 24-well dishes and cultured in 0.5 ml of medium per well. After cells were treated with each compound for 24 hours, cells were incubated with 5 mg·mL^−1^ of MTT for 3 hours. Blue formazan crystals were solubilized with acidified isopropanol, and formazan levels were determined at 570 nm using a spectrophotometer.

### Immunoblotting and immunoprecipitation

Total proteins were separated on an SDS polyacrylamide gel and transferred to Immobilon-P membranes (Millipore, Bedford, MA). Membranes were blocked with 5% nonfat milk in a 0.1% Tween-20/Tris-buffered saline (TTBS) at room temperature for 1 hour, and sequentially incubated overnight at 4°C with a primary antibody (1:1000) and with horseradish peroxidase-conjugated secondary antibody (1:5000) for 1 hour. The immune complexes were visualized using an Enhanced Chemiluminescence Plus kit (Amersham Biosciences, Piscataway, NJ). The intensities of HIF-1/2α blots were quantified using ImageJ program v1.31 (NIH, Bethesda, MD) and normalized to β-tubulin levels. Data from 3 independent experiments were compared statistically. To analyze the interaction between HIF-1α and ARNT, cell lysates were incubated with anti-HIF-1α antibody for 4 hours, and further incubated with protein A/G-Sepharose beads (GE Healthcare BioSciences, Westborough, MA) at 4°C for 2 hours. Co-precipitated ARNT was identified by immunoblotting with anti-ARNT antibody.

### Immunofluorescence

A549 cells were cultured on glass coverslips. The cells were fixed with 4% paraformaldehyde for 30 minutes, washed twice with PBS, blocked with 5% FBS, and incubated with anti-HIF-1α antibody for 16 hours. The cells were incubated with anti-rabbit IgG antibody conjugated with Alexa Fluor^®^ 488 for 1 hour, and their nuclei were stained with 30 μM DAPI for 5 minutes. Finally, the cells were mounted with the Fluorescence Mounting Medium (Thermo Fisher Scientific, Waltham, MA) and photographed under a fluorescence microscope.

### Secreted VEGF assay

A549 cell was treated with a compound under normoxia and hypoxia for 24 hours. VEGF released from cells was analyzed in medium using the human VEGF ELISA kit provided by Life Technologies. The VEGF levels were calculated based on the standard curve using a recombinant human VEGF165. Each assay was triplicate to reduce the intra-assay error.

### Quantitative RT-PCR

Total RNAs were reverse-transcribed, and real-time polymerase chain reaction analyses were performed in 96-well optical plates. Data were analyzed using CFX Manager Software (Bio-Rad Laboratories, Hercules, CA), and values were normalized to β-actin. The sequences (5′ to 3′) of primers are ACCAAGGGACATCAGATTTC & GTAGCCATAGTCACAGGATG for lysyl oxidase (LOX); GGAAGAAAACAGTGCCTATG & GTGTAGTCAGAG ACCCCTCA for carbonic anhydrase IX (CA9); ACCC ACCTCCCTTATGTAGT & CCAACTAATGCAATTT TTCC for CBP/p300-interacting transactivator with G/D-rich carboxy-terminal domain 2 (CITED2); GGAG GAGAACAAACAGATCA & GTAGGACAGGAAGTTG TTGG for cyclin D1; GTTGCCACTTCCACATAATG & TCATCTGTGCTTTCATGTCA for HIF-1α; GTGGACA TCTTCCAGGAGTA & GTGATGTTGGACTCCTCAGT for vascular endothelial growth factor (VEGF); and ggacctgactgactacctca & agcttctccttaatgtcacg for β-actin.

### Anchorage-independent tumor growth assay

The top agar mixture (RPMI-1640 with 10% fetal bovine serum, 0.4% agar, and 2 × 10^3^ A549 cells) was poured into a dish which had been coated with 0.8% agar, and solidified. Cells were incubated for 3 weeks, and fresh media containing compounds were administered twice a week. Colonies were identified and calculated under an inverted microscope at ×100 magnification.

### Endothelial tube formation assay

Endothelial tube formation assay was conducted on the Matrigel (BD Biosciences, San Jose, CA), which was added to a 24-well plate in a volume of 100 μL/well and allowed to be polymerized at 37°C for 30 minutes. Human umbilical vein endothelial cells (HUVECs) at a density of 2 × 10^4^ were seeded into each well and incubated with 0.5 mL of MEDIUM 199 (Sigma-Aldrich) containing 1% FBS. After stabilized, cells were incubated in the conditioned media driven by A549 cells for 16 hours. Images were captured with OLYMPUS camera attached to the microscope. The tube formation was quantified by measuring the long axis of the individual cells using ImageJ.

### Animals and tumor grafts

Male nude mice (BALB/cAnNCrj-nu/nu) were purchased from Charles River Japan (Shin-Yokohama, Japan) and housed in a specific pathogen-free room. Mice (11 weeks old) were injected subcutaneously in a flask with 1 × 10^6^ LLC1 cells. After tumor volumes reached over 100 mm^3^, mice were intraperitoneally injected with dimethyl sulfoxide (DMSO) or DAS (1 mg kg^−1^) once a day. Tumor volumes were measured with a caliper and calculated using the equation “a × b^2^/2”, where ‘a’ is the maximal width and ‘b’ is maximal orthogonal width. All animal procedures were performed in accordance with a protocol approved by the Institutional Animal Care and Use Committee (Approve #SNU-141008-1).

### Immunohistochemistry

Xenograft tumor tissue specimens were deparaffinized, autoclaved to retrieve antigens, and sequentially incubated with 3% H_2_O_2_, a primary antibody (anti-HIF-1α; anti-CD31, Thermo Fisher, Rockfordd, IL); anti-Ki67, Leica Biosystems, Richmond, IL), and a biotinylated secondary antibody (Vector Laboratories, Burlingame, CA). The immune complexes were visualized using Vectastatin ABC (Vector Laboratories), and the specimens were counterstained with hematoxylin. Histology and immunostaining were reviewed under a microscope at a ×400 magnification and all slides were reviewed independently by four parts.

### Statistical analyses

Statistical analyses were performed using the Microsoft Excel (2010) software and values were described as means and standard deviation. The differences between two groups were analyzed by two-sided, unpaired Student *t-test*, and were considered statistically significant when *P* < 0.05.
